# Volunteer Community Health and Agriculture Workers Help Reduce Childhood Malnutrition in Tajikistan

**DOI:** 10.9745/GHSP-D-20-00325

**Published:** 2021-03-15

**Authors:** Roman Yorick, Faridun Khudonazarov, Andrew J. Gall, Karah Fazekas Pedersen, Jennifer Wesson

**Affiliations:** a IntraHealth International, Dushanbe, Tajikistan.; b IntraHealth International, Chapel Hill, NC, USA.

## Abstract

Childhood malnutrition is a nationally-recognized problem in Tajikistan. In 2017, 6% of children under 5 years were wasted and 18% were stunted. Through the Tajikistan Health and Nutrition Activity (THNA), funded by the U.S. Agency for International Development's Feed the Future, IntraHealth International trained 1,370 volunteer community health workers (CHWs) and 500 community agricultural workers (CAWs) in 500 rural communities to improve nutrition among children and pregnant and breastfeeding women. CHWs and CAWs mutually encourage health behavior change, reinforce better agricultural practices, and promote maternal and child health and nutritious diets through household visits, community events, and peer support groups. CHWs refer children with malnutrition and diarrhea and pregnant women who are not registered for antenatal care to health facilities. THNA supported CHWs/CAWs through peer learning, refresher trainings, supportive supervision, and quarterly material incentives. We observed gains in knowledge, attitudes, and practices across health; nutrition; water, sanitation, and hygiene (WASH); and agriculture in target communities. From 2016 to 2019, we observed statistically significant (*P*<.05) improvements in children receiving a minimum acceptable diet; children with diarrhea receiving more liquids; women making 4 or more antenatal care visits; women reporting improved WASH; and farmers demonstrating improved agricultural practices. A February 2020 screening of 94.6% of children under 5 years in target communities found the prevalence of children with signs of wasting at 2.2%. Partnerships between CHWs, CAWs, and rural health workers facilitated these results. Paired agricultural and health interventions proved successful in improving nutrition of children and may be applicable in other contexts. Although effective in delivering interventions, CHWs/CAWs experience attrition, need motivation, and require intensive support. Assuming responsibility for this community-based volunteer workforce presents a major challenge for Tajikistan's national and local governments.

## Abstract (precis)

Paired agricultural and health interventions led by volunteer community health workers and community agricultural workers through home visits, community events, and peer support groups proved successful in improving nutrition of children and may be applicable in other contexts.

## Teaser

**Key Findings:**

- Through social and behavior change communication activities, this nutrition-specific and nutrition-sensitive project that included interventions in maternal, newborn, and child health (MNCH); water, sanitation, and hygiene (WASH); and agricultural practices improved dietary diversity of women and children in Tajikistan.
- Community volunteers specializing in agriculture or MNCH and WASH are an effective workforce for improving individual knowledge, attitudes, and practices that result in better nutrition.
- Demonstrating sustainable improvement in children's nutritional status over a 5-year project presents a significant challenge.

**Key Implications:**

- Policy makers and donors should consider long-term multifaceted approaches to improving nutrition of women and children that include health, agriculture, and WASH interventions.
- Program managers should consider community volunteers as an effective change instrument that requires continued technical support and motivation, as well as training to replace dropouts.
- Implementers should be ready to adapt their interventions to address local family structure, power dynamics, beliefs, traditions, and myths surrounding nutrition of women and children.

## BACKGROUND

Malnutrition has life-threatening, lifetime, and generational consequences for children in low- and middle-income countries, where almost half of all children under 5 years reside. Globally, 65% of children who are stunted and 73% of those who are wasted live in these countries.^1^ In Tajikistan, the most impoverished country in Central Asia, childhood malnutrition is a nationally recognized problem, compounded by anemia in women of reproductive age and children under 5 years and diarrhea due to poor water, sanitation, and hygiene (WASH). Although much progress has been made in recent years in the nutritional status of children in the country, 6% of children under 5 years were wasted in 2017 (down from 10% in 2012) and 18% were stunted (down from 26% in 2012).^2^^,^^3^

Rural farming communities in Tajikistan are disproportionately poorer, more food insecure, and undernourished compared to other communities.^3^ Khatlon province—where rural farming communities make up 83% of the population of approximately 3,274,900^4^—has the highest rates in the country of under-5 mortality (40 per 1,000 live births in 2017; down from 61 in 2012) and stunting (19% in 2017, down from 27% in 2012).^2^^,^^3^ In addition, 25% of women in Khatlon are not achieving dietary diversity (compared to 20% nationally); 47% of women are anemic (41% nationally) and 46% of young children aged 6–23 months are anemic (42% nationally).^3^

Pathways to preventing and mitigating malnutrition include adequate maternal nutrition before and during pregnancy and lactation; optimal breastfeeding in the first 2 years of life; access to and consumption of nutritious, diverse, and safe foods; a healthy environment, including access to basic health care and WASH; and opportunities for safe physical activity.^1^ To address these pathways, evidence to date suggests long-term integrated, multisectoral programming that is both nutrition specific and nutrition sensitive are needed.^1^^,^^5^ Programmatic efforts that integrate agricultural and community education and behavior change interventions show promise, suggesting greater impacts on child nutritional status when programs incorporate health and WASH interventions, as well as micronutrient-fortified products.^5^^–^^7^ Recent randomized program evaluation studies suggest educational activities that complement agricultural initiatives reflect positive changes to both anthropomorphic measures and childhood anemia.^6^^,^^8^ However, a series of reviews on agricultural interventions' effect on nutrition outcomes for children, such as stunting, wasting, or underweight, showed mixed or inconclusive results,^5^^,^^8^^–^^11^ and measures of dietary diversity among women or children tended to increase.^9^^–^^11^ Several reviews have suggested that mixed or inconclusive results were due to weak study designs and evaluations.^10^^,^^12^

Programs that integrate agricultural and community education and behavior change interventions show promise on impacting child nutritional status.

**FIGURE 1:**
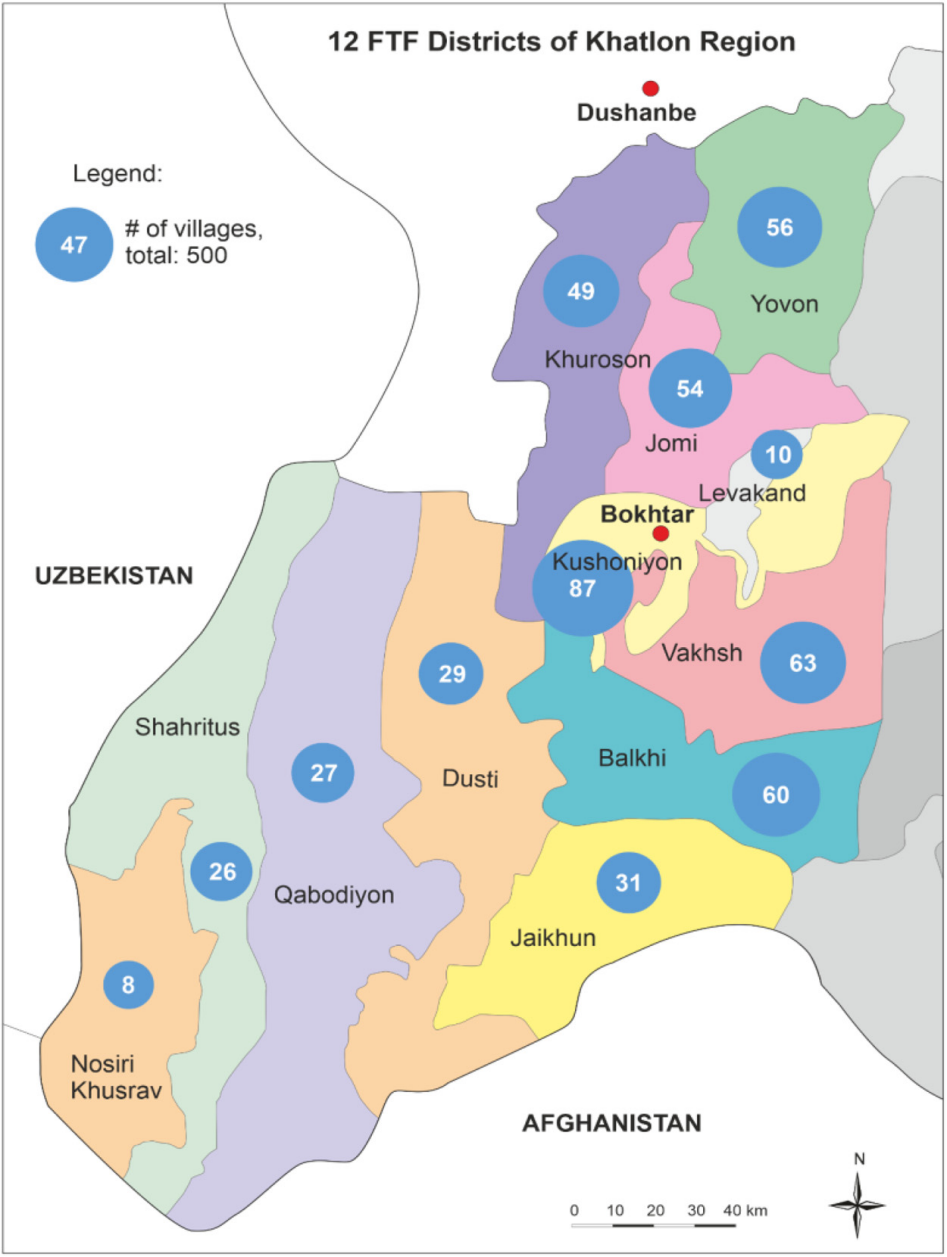
Tajikistan Health and Nutrition Activity Target Villages in 12 Districts, Khatlon Region, Tajikistan Abbreviation: FTF, Feed the Future.

The Tajikistan Health and Nutrition Activity (THNA), funded by the U.S. Agency for International Development's (USAID) Feed the Future and led by IntraHealth International, implemented an integrated community agriculture and health intervention to improve the health and nutrition of women and children in 12 districts of Khatlon (Figure 1). We developed a comprehensive community volunteer strategy based on evidence that equipped, trained, and supported community health workers (CHWs) could promote health and provide high-quality care in remote and poor regions.^13^^,^^14^ THNA trained 1,370 CHWs and 500 volunteer community agricultural workers (CAWs), jointly referred to as community volunteers, in 500 rural communities. Although data are presented starting from 2016, THNA significantly changed its approach to community-based work in 2018. Thus, the article describes THNA's implementation approach from 2018 to June 2020 and uses 2016 data as a baseline.

## PROGRAM DESCRIPTION

THNA addressed underlying and immediate malnutrition factors through an integrated multifaceted approach of working with community volunteers to improve the quality of maternal, newborn, and child health (MNCH) services; increase access to nutritious foods and reduce the burden of infectious and parasitic diseases; and strengthen WASH infrastructure.

### Community Volunteer Engagement and Management

THNA worked with community volunteers to provide MNCH services and nutrition or agricultural education and serve as change agents for the following reasons: (1) behavior change is best achieved through direct individual or small-group interventions; (2) the project's large number of target districts, communities, and individuals; and (3) the shortage of other change agents, such as government health providers or local nongovernmental organizations. THNA conducted comprehensive selection and training processes and supported volunteers in their work through monthly peer-learning sessions at the district level and regular supportive supervision visits at the community level based on the volunteers' needs.

#### Selection and Training

THNA transitioned some community volunteers from a previous community-based project (2011–2015) where they worked as “community health educators” on both MNCH and agricultural topics. There were no other volunteer-based activities in THNA districts before 2011. THNA offered to retrain “community health educators” either as CHWs or CAWs and recruited additional community volunteers to increase coverage. Criteria for community volunteer recruitment included: at least secondary school education; self-motivation and interest in community volunteer work; ability to devote at least 8 hours a week; good networking and communication skills; positive relationships with neighbors, village leaders, and health providers; not being currently employed as a health worker (for CHWs); and availability to travel 1 day a month to the district center for peer-learning meetings. Women made up the majority of THNA community volunteers (89%), with a median age of 46 years.

THNA provided an initial training for CHWs over 5 days on all relevant topics and trained CAWs for a total of 8 days, 2 days per quarter, on seasonal topics relevant to the upcoming agricultural season (Table 1). All volunteer trainings were participatory, combining development of practical skills with acquisition of new knowledge. We assessed trainees' knowledge through pre- and post-tests; those achieving at least 80% on the post-test received certificates of achievement, those who did not pass had the option to take the course again. Initially, THNA had an annual attrition rate of 17%. With the addition of repeated training courses and clearer expectations on time and responsibilities, the attrition rate of CAWs and CHWs dropped to 1% in the last project year.

**TABLE 1.** THNA Community Volunteer Training Topics, Khatlon Region, Tajikistan

Community Health Worker Training	Community Agricultural Worker Training
Communication skills and principles of adult learning Safe WASH principles Principles of healthy nutrition, including micronutrients Exclusive breastfeeding and complementary feeding of children Childhood malnutrition; child growth monitoring and promotion Prevention of anemia in women and children Antenatal care, nutrition, and danger signs during pregnancy Conducting household visits, and THNA data collection requirements Cooking demonstrations Facilitating and leading peer support groups on various topics	Fall Communication skills and principles of adult learning Poultry care and dairy production (home cheese-making) Winter Household budgeting Family entrepreneurship Spring Home garden management: Crop rotation Disease management Irrigation Pest management Soil fertility (composting) Summer Post-harvest technologies (food preservation and storage)

Abbreviations: THNA, Tajikistan Health and Nutrition Activity, WASH, water, sanitation, and hygiene.

By adding repeated training courses and clearer expectations on volunteer workers' time and responsibilities, THNA's annual attrition rate dropped from 17% to 1%.

#### Monthly Peer-Learning Sessions

THNA conducted more than 40 peer-learning sessions, with 20–25 community volunteers each, separately for CHWs and CAWs, at the end of every month. The goals of these sessions included sharing of successes and challenges, finding solutions to common problems, testing knowledge through quizzes and providing refresher trainings based on the results, teaching new knowledge and skills through interactive training sessions, analyzing results of the previous month's work, and collecting data reports on the current month. Select rural health workers, village leaders, and staff of government public health institutions (healthy lifestyle centers) also participated in these meetings.

#### Supportive Supervision Visits

THNA staff selected villages and volunteers for supportive supervision based on achievements. Valid high achievements deserved analysis and potential sharing of “golden nuggets” with other volunteers. Low achievements necessitated support to address challenges and improve performance. Such challenges included lack of support from the village head or government health worker and volunteers' poor communication skills or lack of experience. Other volunteers also received supportive supervision visits on an as-needed basis.

#### Incentives for Community Volunteers

At each meeting, THNA staff recognized individual volunteers for high achievements in the previous month (e.g., number of children with malnutrition identified, number of households visited) and awarded them small nonmonetary prizes, up to US$5 in value. Over the years, THNA organized 2 cross-district competitions among volunteers to determine and recognize those with the strongest knowledge and skills and highest achievements. The project recognized district and regional winners with certificates and small nonmonetary awards. All community volunteers, regardless of their achievements, received a quarterly material incentive worth about US$15 (e.g., umbrellas, blankets, and steam pots).

### Health and Nutrition and Agriculture Interventions

#### Household Visits

Household visits provided the main mode of community volunteer activity. On average, each CHW visited 20 households per month, and each CAW visited 25 households, specifically targeting households with children under 2 years and children with malnutrition or other known medical conditions; low-income households and incomplete families; and households with pregnant women and with newlyweds to counsel them on antenatal care and nutrition of pregnant women. During visits, community volunteers provided individual or small group educational sessions and practical training on MNCH, nutrition, WASH, or agricultural topics relevant to the specific household, using visual aids, videos, and information materials. CHWs also identified and referred pregnant women not registered for antenatal care and children with signs of malnutrition and/or with diarrhea to primary health centers. CHWs routinely screened children for signs of malnutrition using mid-upper arm circumference (MUAC) tapes. A total of 1,852 children were referred over 18 months in 2018–2019.

Although household visits were the main community volunteer activity, other health and nutrition interventions included community events, health fairs, cooking demonstrations, campaigns, and peer support groups.

#### Child Growth and Promotion Campaign

The 2017 Demographic and Health Survey (DHS) found 6% of children under 5 years wasted in THNA target districts.^3^ To gauge the current child nutritional status in target communities, in February 2020, THNA organized a child growth and promotion campaign in which CHWs and CAWs partnered with government health workers to identify true prevalence of malnutrition through a cross-sectional survey of all children aged 6–59 months. All community volunteers, as well as rural health workers, received training in using MUAC tapes. Over a month, they conducted household visits and measured 94.6% of 111,313 children under 5 years registered with health facilities, identifying 2,437 children (2.2%) with signs of malnutrition.

#### Small Group Practical Training on Agricultural Topics

Each CAW reached an average of 140 individuals every month with small group trainings on seasonally appropriate agricultural topics. CAWs provided their training during household visits, lunch breaks during field work, community events, or work projects for public good, such as removing garbage or cleaning an irrigation or drainage channel.

#### Cooking Demonstrations

In Tajikistan, many beliefs and prejudices prevent pregnant women, breastfeeding women, and children aged 6 months and older from receiving nutritious foods. CHWs and CAWs jointly facilitated at least 1 cooking demonstration a month to promote age-appropriate nutritious recipes from the THNA recipe book, as well as WASH principles. Each demonstration covered 2 recipes for different target groups and included an educational session on a health and nutrition topic. CAWs also provided sessions on safely preparing home preserves of different fruits and vegetables and preventing botulism.

#### Peer Support Groups

Peer support group meetings, an innovative format in the Tajikistan context, provided a monthly platform for community members to share positive experiences on MNCH and nutrition, reinforce desired behaviors, and find solutions to common challenges. In 2020, CHWs facilitated 624 peer support groups in 491 villages.

#### Community Events

In rural Tajikistan, traditions of folk theater are very strong, and dramatic sketches performed by community members are quite popular. To raise awareness and education on nutrition and related topics, THNA staff, CHWs, and CAWs co-organized larger public events in villages. Taking into account issues most relevant to the host community, THNA staff prepared original scenarios for these events, which included dramatic sketches (role-plays), songs, quizzes, child drawing competitions, and poem recitation. Community events were devoted to WASH and marketing of ventilated improved pit latrines, exclusive and continuing breastfeeding, and marketing of new crops (e.g., broccoli, bok choy, okra, sweet potatoes).

#### Health Fairs

THNA organized health fairs to bring specialty health services into the most remote rural communities, which under normal circumstances only have a nurse and/or a midwife on duty. For these events, THNA provided transportation and disposable supplies for a pediatrician, obstetrician/gynecologist, lab technician, and ultrasound specialist to travel to a remote village and provide services. CHWs invited women and children and provided group and individual MNCH and nutrition counseling for women waiting in line. Health fairs helped register pregnant women for antenatal care and identify women who have pre-eclampsia and children who are malnourished and refer them for care.

#### Referrals and Linkages

THNA worked in partnership with the regional government department of health and its rural health facilities present in 337 of 500 target villages. CHWs referred pregnant women, children with signs of malnutrition, and children with diarrhea to local health facilities. In turn, government health workers referred women and children to CHWs for community follow-up and care. CHWs met with rural health providers once a month or more often, if needed, to reconcile the list of referred clients and coordinate activities.

## METHODS

To assess the results and adjust the course of community-based interventions, THNA conducted 3 rounds of a recurring agricultural practices survey (RAPS) and 5 rounds of a recurring household survey (RHS). RAPS and RHS used the longitudinal sentinel community surveillance methodology^15^ to detect changes in knowledge, attitudes, and practices over time through repeated cycles of household interviews in select “sentinel” communities.

### Recurring Agricultural Practices Survey

The RAPS assessed exposure to THNA activities and the change in self-reported and observed agricultural practices in 10 villages through 3 annual surveys (September 2017, 2018, and 2019). THNA purposively selected the villages to represent communities from each of the 4 geographic subregions of its Feed the Future zone of influence (ZOI). Within each village, interviewers randomly selected households to reach 100 per district, for a total sample size of 400 households.

THNA selected and trained interviewers from among its staff and CAWs (who were not deployed to the districts in which they worked). The interviewers randomized households in every community by flipping a coin to choose the direction on a street and then going into every fourth household. THNA used a geopositioning service to record daily movements of the interviewers and advise on sections of the village that were not covered. Exposure to THNA agricultural activities was the only eligibility criterion.

Interviewers used a structured interview tool installed on a tablet and conducted interviews in Tajik or Uzbek, depending on respondents' language preference. Data from the tablet were uploaded to an online Kobo Collect database later when a wireless Internet connection was available. We analyzed data in MS Excel (Office 365 version) and STATA (15.1). We calculated statistical significance of observed differences, between round 1 and round 3 in surveyed villages (baseline-endline analysis). To determine statistical significance, we used a conservative logistic regression model to avoid false positives.

### Recurring Household Survey

The RHS followed a panel of 4 THNA communities, collecting data every 6 months (rounds 1–4 in 2016–2018) and a year later (round 5 in 2019). THNA purposively selected the 4 villages at the beginning of the project to represent communities from each of the 4 geographic subregions of the ZOI. In round 5, THNA added 2 comparison communities outside of the ZOI in districts with similar socioeconomic characteristics and no prior or concurrent interventions implemented similar to THNA. Selection criteria included the perceived economic status of the district (3 “better-off” and 3 “poorer” districts), the size of the village (200–250 households), and the distance to the district's center (40–50 km). Within each village, interviewers randomly selected 60 households, for a total sample size of 240 households in the THNA districts and 120 households in the comparison districts.

Respondents included mothers aged 18 years or older, who had participated in THNA activities, and who had at least 1 child under 5 years present in the household at the time of the survey. If more than 1 woman in a household met selection criteria, the interviewer randomly selected the woman to be interviewed by flipping a coin. For anthropometry measurements, interviewers measured all children under 5 years of the selected mother. When asking questions about behaviors and practices, the interviewers focused on the youngest child.

The interviewers used structured questionnaires to assess respondents' knowledge, attitudes, and self-reported practices regarding MNCH, nutrition, and WASH. Interviews were conducted in Tajik or Uzbek depending on the respondents' preference. We analyzed data in MS Excel (Office 365 version) and STATA (15.1). We calculated statistical significance of observed differences, first between round 1 and round 5 in intervention villages (baseline-endline analysis), and second between round 5 comparison villages and intervention villages (control-treatment analysis). We used a conservative logistic regression model to avoid false positives.

## PROGRAM RESULTS

### Survey Participants Demographics

The 2 samples were different based upon the design of the survey (Table 2). RAPS participants could be men, but the vast majority were women (96%, 88%, 95%, respectively). All of the RHS respondents were women. The age groups were more evenly distributed in the RAPS sample. One-third of the RHS respondents were aged 18–29 years and two-thirds were aged 30–44 years, which can be explained by the selection criterion that the woman had to have a child under 5 years. There were no differences in the characteristics of the women in the intervention and comparison communities.

**TABLE 2.** Demographics of Recurring Agricultural Practices Survey and Recurring Household Survey Respondents, Khatlon Region, Tajikistan

	Respondents' Age					
	18–29 Years, %		30–44 Years, %		45 Years and Older, %	
	Female	Male	Female	Male	Female	Male
**Recurring Agricultural Practices Survey**						
September 2017 (N=400)	31	18	40	47	29	35
September 2018 (N=360)	17	2	43	19	41	79
September 2019 (N=404)	19	5	41	16	40	79
**Recurring Household Survey**						
**Intervention Group**						
October 2016 (N=242)	70		30		0	
May 2017 (N=240)	71		29		0	
November 2017 (N=240)	66		34		0	
June 2018 (N=249)	69		30		0	
June 2019 (N=244)	67		33		0	
**Comparison Group**						
June 2019 (N=120)	66		33		1	

### Improved Agricultural Technology

**FIGURE 2:**
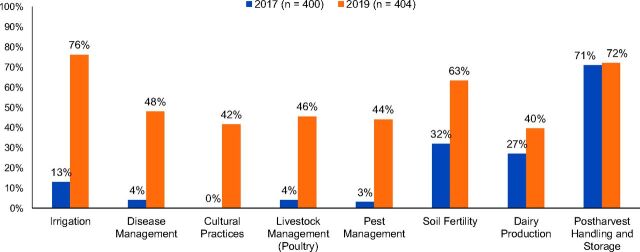
Percentage of Recurring Agricultural Practices Survey Respondents Applying Improved Agricultural Technologies, Between 2017 and 2019, Khatlon Region, Tajikistan

According to the RAPS, nearly all respondents (96%–97%) were exposed to information on post-harvest handling and storage of fruits and vegetables, but there was virtually no change over time in its application (Figure 2). Between 2017 and 2019, we observed a moderate increase in the coverage of topics on home cheesemaking and soil fertility (composting). The biggest increases (*P*<.01) occurred in coverage on the topics of irrigation, cultural practices (crop rotation), disease and pest management, and on livestock management under the poultry care and vaccination topic. The self-reported use of improved agricultural practices remained high throughout the 3 survey rounds, between 93%–100%.

Application of at least 1 improved agricultural practice, as confirmed by interviewer observation, increased from 84% in 2016 to 100% in 2019. Significant increases (*P*<.01) in the application of improved agricultural technologies were found in irrigation, disease management and pest management, livestock management (poultry care), cultural practices (crop rotation), and in soil fertility (composting, *P*<.05) (Figure 2). Moderate but not significant improvements were found in dairy production (cheesemaking).

The percentage of respondents applying multiple improved agricultural practices also increased over time. In 2017, we found 29% of respondents had applied more than 2 improved agricultural practices, and none had applied more than 6 practices. In 2019, 76% of respondents applied more than 2 improved practices, and 30% applied more than 6.

### Feeding Practices and Dietary Diversity

**FIGURE 3:**
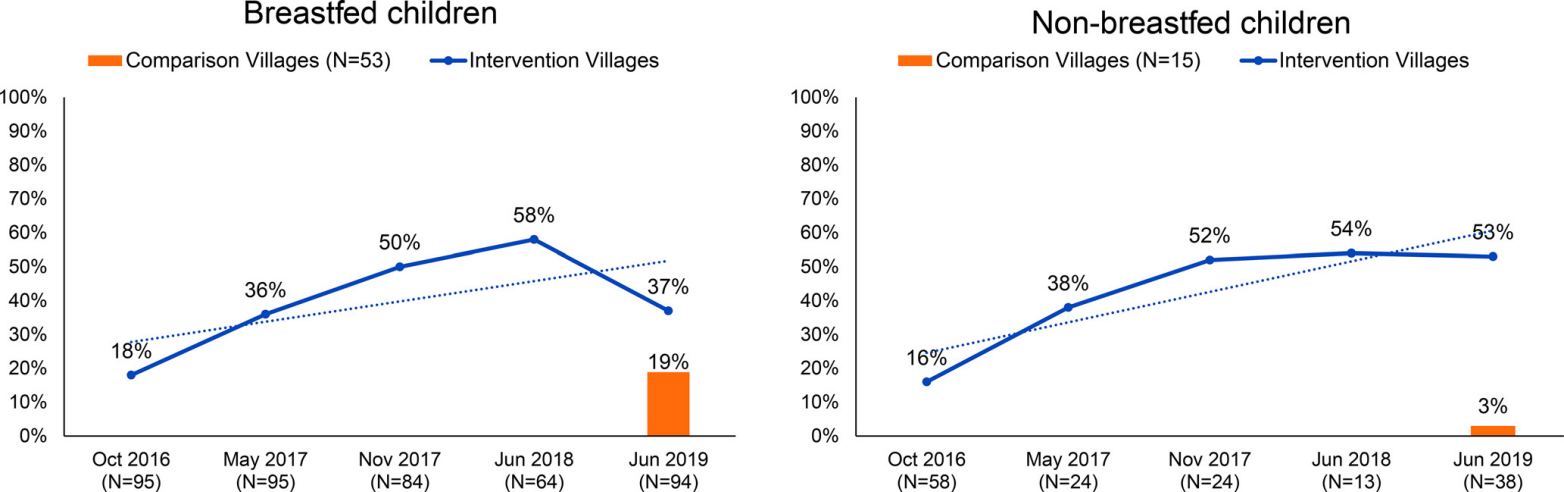
Percentage of Children Who Achieved Minimal Acceptable Diet Among Recurring Household Survey Respondents, Between 2016 and 2019, Khatlon Region, Tajikistan

The proportion of children with minimum acceptable diets increased 2-fold for breastfed children and 3-fold for non-breastfed children, despite having started at very similar proportions (Figure 3). Breastfed children lost ground between June 2018 and June 2019. The differences between intervention and comparison villages were dramatic both for breastfed and non-breastfed children and significant for the non-breastfed group (*P*<.05). For non-breastfed children, the result was statistically significant for both the baseline-endline and control-treatment analysis. Table 3 shows comparisons of key RHS data with DHS 2017 data for feeding patterns and other indicators.

**TABLE 3.** Recurring Household Survey Data Compared to Tajikistan Demographic Health Survey Data, Khatlon Region, Tajikistan

		Recurring HouseholdSurvey: Round 1 (October 2016)	Recurring Household Survey: Round 5 (June 2019)		Demographic Health Survey (2017)
			Comparison Villages	Intervention Villages	
**Feeding Practices and Dietary Diversity**					
Exclusive breastfeeding 0-5 months, %		-	50	72	36
Continuous breastfeeding, %		68	78	71	50
Minimum acceptable diet in children aged 6–23 months, %	Breastfed	18	19	37	9
	Non-breastfed	16	3	53^a,b,c^	10
Child feeding practices during diarrhea, %	More fluids	33	63	80^a,b^	27
	More food	36	21	55	10
Women achieving minimum dietary diversity, %		84	71	90	80
**Water, Sanitation, and Hygiene**					
Households with soap present at handwashing station, %		48	68	70	76
Soap use after defecation, %		24	37	88^a,b^	-
Soap use after cleaning a child, %		19	65	80^a,b^	-
Soap use before feeding a child, %		22	25	69^a,b^	-
Soap use before preparing food, %		21	45	82^a,b^	-
Soap use before eating, %		24	8*	78^a,c^	-
**Health Seeking Behaviors**					
Number of antenatal care visits for previous pregnancy		3.6	4.0	5.7	-
Women who had 4+ antenatal care visits, %		57	48	86^a,b^	64
Women participating in their health care decision making, %		4	4	11	-
**Knowledge on Women and Children's Nutrition**					
Women who know of the need of a pregnant woman to eat more, %		64	49	89^a,b,c^	-
Women who know that breastfeeding women should eat more, %		77	66	99^a,b,c^	-
Women who know that a baby should receive more breastmilk during diarrhea %		44	28	93^a,b,c^	-

a Results are statistically significant at *P*<.05, utilizing a conservative logistic regression model.

b Result is significant compared to Round 1 (baseline/endline analysis).

c Result is significant compared to comparison villages (treatment/control analysis).

Women's nutrition practices did not improve as significantly as those of the children, but there was some degree of improvement. Between 2016–2019, women's minimum dietary diversity increased slightly from 84% to 90%. In 2019, women's dietary diversity was 19 percentage points higher in intervention villages than in comparison villages (71%), but it was not a statistically significant difference. However, THNA data on minimum dietary diversity in women are still higher than those of DHS 2017 for the ZOI districts (80%). There was little change in the average number of food groups consumed between 2016 (6.2) and 2019 (6.7).

The percentage of women who reported giving more fluids to a child with diarrhea in the 2 weeks preceding the survey increased significantly (*P*<.02) between 2016 (33%) and 2019 (80%). The percentage of women who reported giving a child more or the same amount of food during diarrhea also increased (2016, 36%; 2019, 55%), but the improvement was not statistically significant. The increase in fluids was statistically significant for the baseline-endline analysis. The increase in food was not statistically significant in either analysis. Both of these indicators are much higher in THNA target villages compared to DHS 2017 data (27% for increased fluids and 10% for increased food).^3^ Our measurements of exclusive breastfeeding practice were flawed and inconsistent, so we are unable to analyze that data with rigor and report on that topic. In Tajikistan's hot climate, the belief in the need of breastfed children for additional liquids is prevalent and persistent even among government health workers. In the first 3 RHS rounds, the question on exclusive breastfeeding did not specify that it excludes additional liquids, which yielded a very high prevalence of exclusive breastfeeding. In the last 2 RHS rounds, THNA clarified the survey question and received more realistic results.

### Water, Sanitation, and Hygiene

**FIGURE 4:**
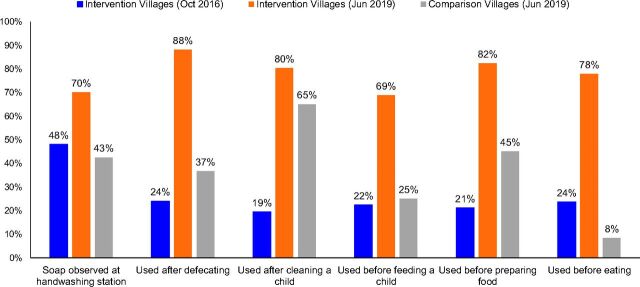
Direct Observation of Soap at Handwash Stations and Self-Reported Use of Soap, Among Recurring Household Survey Respondents, Between 2016 and 2019, Khatlon Region, Tajikistan

The percentage of women who reported washing hands with soap after defecation (*P*<.01), after cleaning the child (*P*<.01), before feeding the child (*P*<.05), before preparing food (*P*<.05), and before eating increased between 2016 and 2019 (Figure 4). Women in THNA villages reported washing hands with soap more frequently than women in comparison villages, but the only statistically significant difference was in using soap before eating (*P*<.01).

Observed presence of soap at handwashing stations may be influenced by seasonal factors. After the start of the rainy season (October-November), households may not keep soap at handwashing stations that are outdoors. If seasonality is excluded (considering only May-June), there is no identifiable trend in the presence of soap. In 2019, interviewers observed soap less often than would be expected from the proportions of households claiming to be using soap for nearly every category of soap use. In comparison villages in 2019, soap was present less often than in intervention villages, although the difference was not statistically significant based on our analyses.

### Health-Seeking Behaviors

Between 2016 and 2019, we observed an upward trend in antenatal care visits by women in intervention villages. The average number of visits increased from 3.6 to 5.7, which is an improvement, but still short of the 7 antenatal care visits that are recommended by Tajikistan's national guidelines. The percentage of women who attended at least 4 antenatal care visits increased from 57% in 2016 to 86% in 2019. Antenatal care attendance in 2019 was significantly higher in intervention villages than comparison villages according to our treatment-control analysis (86% vs. 48%, *P*<.05).

### Knowledge on Women's and Children's Nutrition

Between 2016 and 2019, the percentage of respondents in intervention villages who knew that women should eat more during pregnancy increased from 64% to 89% (*P*<.05). The percentage of respondents who knew that breastfeeding women should eat more than usual increased from 77% to 99% (*P*<.05). Respondents also improved knowledge on breastfeeding babies during diarrhea episodes, an improvement from 44% to 93%. Respondents in intervention villages had statistically significantly higher knowledge in all these categories than respondents in comparison villages.

## DISCUSSION

THNA combined community-based agricultural, MNCH, nutrition, and WASH interventions using community volunteers, a unique model in the Central Asia region and other low- or middle-income countries. The fact that all community-based interventions were implemented by community volunteers was key given the large number of target districts, villages, and beneficiaries and the shortage of alternative social and behavior change communication conduits, such as nongovernmental organizations or government health workers. Village communities in the focus districts responded well to individual and small group social and behavior change communication interventions implemented through the volunteers, as evidenced by the large number of community contacts and the changes we observed in behaviors. A recent global evidence review of nutrition-sensitive agricultural interventions found just 2 rigorous evaluations of interventions that included behavior change elements led by community volunteers.^5^ Both of these interventions demonstrated promising effects on child nutrition and other indicators, although neither could demonstrate positive changes in addressing stunting among children, similar to other nutrition-sensitive interventions examined in the review.^5^ Given THNA's results in using a community volunteer model and the limited number of similar interventions in the literature that nevertheless showed promising results, more evaluations of carefully designed integrated, multisectoral community-led or community volunteer agriculture, nutrition, and health interventions that leverage existing community health structures in resource-constrained settings are needed.

Village communities in the focus districts responded well to individual and small group social and behavior change communication interventions implemented through the volunteers.

A major challenge for THNA was engaging men in MNCH and nutrition issues. In traditional Tajik families, men are the main breadwinners, and many of them travel within the country or abroad for work. Younger women (unmarried daughters and daughters-in-law) complete most household chores, provide childcare, and assist men in field work. It is mostly older women who have the time to devote to community volunteer work. Older women (mothers-in-law) are the ultimate decision makers in their families, particularly when men are away for work. The high proportion of older women who served as community volunteers (53% were aged 45 years or older) strengthened the influence and social status of volunteers. We learned that all community-based activities had to start by engaging older women. Without their buy-in and support, participation of younger women would not be allowed, including speaking to volunteers during household visits or attending cooking demonstrations or peer support groups.

*I am grateful to the project, because you changed the attitude of my mother-in-law! Before, she was stingy and would not let me eat enough, and was angry when I spent too much time breastfeeding. Now, she started caring for my health and nutrition, lets me breastfeed the child as long as necessary, and allowed me to participate in cooking demonstrations and peer support group meetings.* —Daughter-in-law

Peer support groups—a novel and innovative concept in Tajik villages—were designed to highlight positive behaviors; demonstrate to other group members the successes of their fellow village members in antenatal care, exclusive breastfeeding (EBF), complementary feeding, and other topics; and support group members by sharing experiences in overcoming challenges.

Volunteers shared that their families expect them to bring something home in return for the time and effort spent on community work. We believe the incentives we provided are part of the reason for our low attrition rates. At the same time, the volunteers' main motivation was the status they received in their villages. This included the ability to travel once a month to the district center for an important meeting or having an unofficial title of “doctor's assistant” in the village.

Despite their relatively small number (1 per village regardless of its size), CAWs demonstrated high coverage of beneficiaries with information and demonstrations of improved agricultural practices. As a result, the number of practices applied increased significantly over time. By contrast, CHWs were not able to regularly cover all 100 households they were assigned. Coverage depended on their communication skills and ability to step outside their comfort zone to work with difficult and less welcoming households. By joining forces with CAWs and facility health workers, CHWs were able to overcome some of these barriers. Additional training on communication skills and allocation of fewer households per volunteer may be necessary to improve their effectiveness.

CAWs demonstrated high coverage of beneficiaries; in contrast, CHWs were not able to regularly cover all 100 households they were assigned, but joining with CAWs and facility workers helped overcome some communication barriers.

Improved agricultural practices, according to THNA's theory of change, increased the variety of foods available to women and children, thus allowing for the increased dietary diversity demonstrated through the RHS. Increased agricultural production might have also contributed to improved diets of women and children through increased income and accessibility of different foods through purchasing. However, we did not assess changes in household income. The use of post-harvest technologies was high at the start of THNA, potentially because of households' exposure to this topic from the previous maternal and child health activity. Most households already applied these technologies, and it was hard to improve on this already high uptake.

CHWs significantly improved the knowledge of women of reproductive age on nutrition of pregnant and breastfeeding women and children. To improve child nutrition, THNA continuously worked on increasing the prevalence of EBF of children aged 6 months or younger, which was at a low 36% nationwide in 2017. Giving children under 6 months clear liquids, such as water or tea, in addition to breast milk is a widespread practice, not usually distinguished from EBF and promoted, even by many pediatricians, due to Tajikistan's hot climate. Because of measurement issues, THNA was not able to demonstrate an improvement in EBF in its target villages. In the first 4 rounds of the RHS, children receiving liquids in addition to breast milk were counted as exclusively breastfed, demonstrating a false EBF prevalence of more than 80%. In the last round of the RHS in 2019, this mistake was corrected, and this last measurement of 72% can be considered accurate. Compared to the 50% in comparison villages, THNA might have made an improvement in EBF in target villages, but the difference was not significant given the size of the comparison group.

Demonstrating an improvement in nutritional status of children under 5 years on a large scale has been challenging for similar projects.^16^ Measurement fluctuations also suggest the influence of some seasonal factors, such as abundant and lean seasons and diarrheal seasons. Similar to other nutrition-sensitive agriculture projects, THNA demonstrated statistically significant improvements in dietary diversity in non-breastfed children aged 6–23 months, and the minimum acceptable diet in non-breastfed children aged 6–23 months. These dietary improvements may be a prerequisite for improvement in the nutritional status of children, which may require more than 4 years of observation to register. Improvements in minimum dietary diversity and minimum acceptable diet in breastfed children were more modest than those in non-breastfed children, potentially because women who did not discontinue breastfeeding after the child had reached 6 months paid less attention to complementary feeding and the diversity of food the child received in addition to breast milk.

Cross-sectional data from the 2020 growth monitoring and promotion campaign by CHWs with the use of MUAC tapes may be a proxy demonstration of the decreased prevalence of wasting in children under 5 years. We found a much lower prevalence of abnormal MUAC measurements than expected given DHS 2017 data on wasting, but the measurement methodologies are very different (MUAC vs. weight and height). Identification rates of children with signs of malnutrition by CHWs during their regular work was lower than those during the growth monitoring and promotion campaign. One of the reasons may be that CHWs were not able to regularly cover all 100 households they had been assigned. Regular growth monitoring and promotion campaigns by community volunteers and government health workers should have been routine practice, but THNA did not test implementation of this campaign approach until the last few months of the project.

THNA used 2 evaluation tools to gauge its progress and adapt its interventions: RAPS and RHS. RAPS was annual, and the RHS was originally designed as a semiannual household survey with 67 questions mimicking those from the national DHS for comparison purposes. However, THNA found semiannual surveys too frequent to analyze the results, revise and pilot adapted interventions, roll out to 12 districts and hundreds of volunteers, and see any changes in 6 months. Therefore, the fifth RHS round was shortened to the most actionable questions (n=46) and switched to an annual schedule.

### Sustainability

To foster continued activities of CAWs and CHWs beyond the project timeline, THNA partnered with the government healthy lifestyle centers (HLSCs), which exist at the national, regional, and district levels. Since 2017, HLSCs are authorized by the Ministry of Health and Social Protection of the Population (MOHSPP) to coordinate the work of CHWs. Until recently, no national framework for recruiting, training, and supporting CHWs existed. To encourage HLSCs' further continuation of THNA activities, HLSC and THNA staff served as cotrainers for THNA's community volunteers. In addition, HLSC staff engaged in THNA program analysis and planning, co-facilitated monthly volunteer peer-learning sessions, and co-facilitated all community-based events. Maintaining these CHW functions beyond THNA's timeline will be a challenge for the government HLSCs given the shortage of resources.

THNA engaged with MOHSPP through the national-level working groups on MNCH and nutrition. THNA advocated for a specific role and responsibilities assigned for community volunteers in the government health system, and additional resources required for HLSCs for facilitating the work of community volunteers. These resources are necessary, at a minimum, for training community volunteers and travel by HLSC staff to the communities for mentoring and supervision. However, the MOHSPP still relies on international donors for supporting community volunteer work. For example, IntraHealth will facilitate the work of the existing CAWs and recruit additional ones within the new 5-year USAID Agriculture and Land Governance Activity, which will work closely with the Ministry of Agriculture.

### Limitations

THNA focused largely on program implementation and lacked the robust evaluation activities to assess the synergistic effects of improved agricultural practices and MNCH and WASH behaviors on children's nutritional status. We originally designed RAPS and RHS as sentinel surveillance to fine-tune project interventions and gauge their effects. The sample design was not sized to detect moderate differences in results. Therefore, RAPS and RHS results are not representative of all 500 target villages or the whole ZOI. To improve our analysis, we added a comparison group to the last RHS round, but the group was not large enough to detect statistical significance of moderate size differences. Most of the practices in the RHS were self-reported, except for the observed availability of soap at handwashing stations; therefore, the results may be skewed to socially desirable responses. RAPS made a distinction between the reported and observed improved agricultural practices to overcome this limitation. The final rounds of RAPS and RHS in 2020 had to be canceled because of the COVID-19 pandemic.

### Applicability to Other Geographical Contexts

THNA's experience may be applicable for improving MNCH and nutrition in traditional agricultural villages facing combined challenges of poverty, low variety of nutritious foods, poor WASH conditions, and hierarchical family structures where women's and children's health and nutrition needs are not high priority. These conditions are present in other countries in Central Asia, Southeast Asia, Africa, and Central America.

## CONCLUSION

THNA demonstrated that volunteer CAWs and CHWs can improve MNCH and nutrition through changing agricultural practices and social behavior in antenatal care, nutrition of women of reproductive age and children under 5 years, and WASH practices.

CAWs and CHWs effectively implemented these interventions with intensive support from a USAID technical assistance project. Such interventions and community volunteer models need to be adapted to the local context. Although successful in delivering interventions, CHWs and CAWs experience attrition, need motivation, and require significant support. Assuming responsibility for this community-based volunteer workforce presents a major challenge for Tajikistan's national and local governments given limited government resources and the ongoing COVID-19 pandemic.

## Notes

**Peer Reviewed**
